# TRPA1 activation leads to neurogenic vasodilatation: involvement of reactive oxygen nitrogen species in addition to CGRP and NO

**DOI:** 10.1111/bph.13519

**Published:** 2016-06-21

**Authors:** Aisah A Aubdool, Xenia Kodji, Nayaab Abdul‐Kader, Richard Heads, Elizabeth S Fernandes, Stuart Bevan, Susan D Brain

**Affiliations:** ^1^ Cardiovascular Division, BHF Centre of ExcellenceKing's College LondonLondonUK; ^2^ Programa de Pós‐graduaçãoUniversidade CEUMASão LuísMABrazil; ^3^ Wolfson Centre for Age Related DiseasesKing's College LondonLondonUK

## Abstract

**Background and Purpose:**

Transient receptor potential ankyrin‐1 (TRPA1) activation is known to mediate neurogenic vasodilatation. We investigated the mechanisms involved in TRPA1‐mediated peripheral vasodilatation *in vivo* using the TRPA1 agonist cinnamaldehyde.

**Experimental Approach:**

Changes in vascular ear blood flow were measured in anaesthetized mice using laser Doppler flowmetry.

**Key Results:**

Topical application of cinnamaldehyde to the mouse ear caused a significant increase in blood flow in the skin of anaesthetized wild‐type (WT) mice but not in TRPA1 knockout (KO) mice. Cinnamaldehyde‐induced vasodilatation was inhibited by the pharmacological blockade of the potent microvascular vasodilator neuropeptide CGRP and neuronal NOS‐derived NO pathways. Cinnamaldehyde‐mediated vasodilatation was significantly reduced by treatment with reactive oxygen nitrogen species (RONS) scavenger such as catalase and the SOD mimetic TEMPOL, supporting a role of RONS in the downstream vasodilator TRPA1‐mediated response. Co‐treatment with a non‐selective NOS inhibitor L‐NAME and antioxidant apocynin further inhibited the TRPA1‐mediated vasodilatation. Cinnamaldehyde treatment induced the generation of peroxynitrite that was blocked by the peroxynitrite scavenger FeTPPS and shown to be dependent on TRPA1, as reflected by an increase in protein tyrosine nitration in the skin of WT, but not in TRPA1 KO mice.

**Conclusion and Implications:**

This study provides *in vivo* evidence that TRPA1‐induced vasodilatation mediated by cinnamaldehyde requires neuronal NOS‐derived NO, in addition to the traditional neuropeptide component. A novel role of peroxynitrite is revealed, which is generated downstream of TRPA1 activation by cinnamaldehyde. This mechanistic pathway underlying TRPA1‐mediated vasodilatation may be important in understanding the role of TRPA1 in pathophysiological situations.

**Abbreviations**
AITC: allyl isothiocyanate
eNOS: endothelial NOS
H_2_O_2_: hydrogen peroxide
iNOS: inducible NOS
nNOS: neuronal NOS
NK: neurokin
NO_2_^−^: nitrite
NO_3_^−^: nitrate
RONS: reactive oxygen nitrogen species
TRPA1: transient receptor potential ankyrin‐1
TRPV1: transient receptor potential vanilloid‐1

## Tables of Links

**Table:** 

**TARGETS**	
**GPCRs** *a*	**Enzymes** *c*
CGRP receptor	Endothelial NOS
NK_1_ receptor	Inducible NOS
**Voltage‐gated ion channels** *b*	Neuronal NOS
TRPA1	
TRPM8	
TRPV1	

**LIGANDS**	
Nitric oxide	1400W
α‐CGRP	Indomethacin
Substance P	NaHCO_3_^‐^
HC030031	L‐NAME
Ruthenium red	Nolpitantium [SR140333]
α‐CGRP‐(8‐37) (human)	

These Tables list key protein targets and ligands in this article which are hyperlinked to corresponding entries in http://www.guidetopharmacology.org, the common portal for data from the IUPHAR/BPS Guide to PHARMACOLOGY (Southan *et al*., 2016) and are permanently archived in the Concise Guide to PHARMACOLOGY 2015/16 (^*a,b,c*^Alexander *et al*., 2015a, 2015b, 2015c).

## Introduction

Primary sensory neurons are widely distributed in the central and peripheral nervous system, and have a physiological role in maintaining vascular homeostasis (Russell *et al*., 2014), although their exact pathophysiological relevance is unclear. Sensory nerve stimulation leads to the release of neuropeptides such as CGRP and substance P, which are established neurogenic‐dependent vasodilators (Brain *et al*., 1985; Lembeck *et al*., 1992). CGRP, in addition to its potent microvascular vasodilator activity, possesses protective mechanisms, which are potentially important for physiological and pathological conditions in the cardiovascular system (Russell *et al*., 2014). We are yet to fully understand the mechanisms involved in the activation of the nerves. The discovery of transient receptor potential ankyrin‐1 (TRPA1), which is located in 60–75% of transient receptor potentials vanilloid‐1 (TRPV1) expressing C‐ and Aδ‐sensory fibres (Story *et al*., 2003; Kobayashi *et al*., 2005) has dramatically increased our understanding of the mechanism underlying activation of the sensory neurons and release of neuropeptides (Bautista *et al*., 2005). Indeed, TRPA1 is expressed on rat sensory nerves (Bautista *et al*., 2005) and human skin (Atoyan *et al*., 2009). It is activated by various exogenous agonists, including pungent extracts such as allyl isothiocyanate (AITC) from mustard oil, cinnamaldehyde from cinnamon and allicin from garlic, which all modify cysteine and lysine residues on the N‐terminus of the TRPA1 channel (Bandell *et al*., 2004; Bautista *et al*., 2005; Hinman *et al*., 2006). Other activators include noxious cold temperature (Story *et al*., 2003; Aubdool *et al*., 2014), products of oxidative stress, lipid peroxidation and hydrogen sulphide (Trevisani *et al*., 2007; Andersson *et al*., 2008; Graepel *et al*., 2011; Eberhardt *et al*., 2014).

The role of TRPA1 in influencing the cardiovascular system is less understood. Deletion of the TRPA1 gene had no effect on blood pressure and cardiac function either under baseline conditions or in response to angiotensin‐II in mice (Bodkin *et al*., 2014). However, systemic administration of the highly selective TRPA1 agonist cinnamaldehyde induced a transient hypotensive response in wild‐type (WT) mice but not in TRPA1 knockout (KO) mice (Pozsgai *et al*., 2010), suggesting that TRPA1 may be involved in regulating the autonomic vasovagal response. Nevertheless, more studies have focused on the role of TRPA1 in regulating the peripheral vascular tone. Whilst the neuronal expression and function of TRPA1 is established, less is known about its vascular expression. TRPA1 is found on the endothelium of cerebral arteries, but not in other vascular beds of rats and humans (Sullivan *et al*., 2015), and AITC mediates an endothelium‐dependent relaxation by activating TRPA1 in rat cerebral arteries (Earley *et al*., 2009). Topical application of mustard oil mediates vasodilatation in the ear skin in a CGRP‐dependent manner (Grant *et al*., 2005; Pozsgai *et al*., 2010), while cinnamaldehyde causes a TRPA1‐dependent vasodilatation in the hindpaw *in vivo* (Pozsgai *et al*., 2010). Recently, we demonstrated that TRPA1 activation by local cold exposure in the hindpaw vasculature induces a transient vasoconstriction followed by vasodilatation, which is essential in returning blood flow to baseline and protecting against local cold‐induced injuries (Aubdool *et al*., 2014). The cold‐induced vasodilator restorative response was mediated by sensory nerve‐derived dilator neuropeptides and NO. This study points to an important role of TRPA1 in regulating peripheral blood flow and a potential target for novel therapeutic approaches (Aubdool *et al*., 2014). However, the critical mechanisms involved in the vasodilator response following TRPA1 activation are unknown.

In the present study, we investigate the mechanisms involved in TRPA1‐mediated vasodilatation using the selective TRPA1 agonist cinnamaldehyde in the mouse ear model *in vivo* by laser Doppler flowmetry. A pharmacogenetic approach allowed us to examine the relative contribution of CGRP and neuronal NOS (nNOS)‐derived NO in cinnamaldehyde‐induced neurogenic vasodilatation. Novel evidence is provided to reveal the pivotal role of reactive oxygen nitrogen species (RONS), especially peroxynitrite generation downstream of TRPA1 activation, with results that highlight a critical role for RONS influencing the neurogenic vasodilatation.

## Methods

*In vivo* experiments were performed according to the UK Home Office Animals (Scientific Procedure) Act 1986 and King's College London Animal Care and Ethics Committee. Animal studies are reported in compliance with the ARRIVE guidelines (Kilkenny *et al*., 2010; McGrath & Lilley, 2015). Male mice were fed *ad libitum* a normal diet and water in a climatically‐controlled environment (22 ± 2°C), maintained under filtered positive pressure ventilation on a 12–12 h dark:light cycle, beginning at 07:00 h. CD1 mice (20–30 g, 8–12 weeks of age, #022, Charles River, UK) and genetically altered mice (8–12 weeks of age) were used, including TRPA1 KO (Kwan *et al*., 2006) (C57BL/6‐B6129P1/F2J mixed genetic background), and WT littermates were bred from heterozygotic mice, which were kindly provided by Drs Kelvin Kwan (Harvard Medical School, Boston, MA, USA) and David Corey (Harvard Medical School, Boston, MA, USA). TRPV1 KO (Caterina *et al*., 2000) and α‐CGRP KO mice (Salmon *et al*., 1999) were raised on a C57/BL6 background, and their WT littermates were used as controls. Up to five mice were housed initially to a cage and then less as they were used for experiments from a minimum of 5 days after delivery to the animal unit. The mouse cages (U‐TEMP Polyetherimide, Techniplast, UK) were 820 cm^2^ by 15.5 cm depth. Bedding was supplied from EcoPure (Nestpak's Aspenchips). All experiments were conducted in a blinded manner, and mice were randomly allocated to treatment groups. The experimenter was blinded towards treatment or genetic background at the time of the experiment. The number of mice used in each group is indicated in the respective figure legends. Mice were anaesthetized i.p. with ketamine (75 mg·kg^−1^) and medetomidine (1 mg·kg^−1^) throughout the experiment (Aubdool *et al*., 2014). At the end of the experiment, all mice were killed by cervical dislocation.

### Measurement of vasoactive responses *in vivo*

Following anaesthesia, cutaneous blood flow was assessed concomitantly in both ears by laser Doppler flowmeter (Moor Instruments Ltd, UK) connected to a PowerLab data acquisition system (AD instruments, UK) whereby a probe, allowing blood flow to be measured precisely at one point in the ear (1 mm^2^ and to 1–2 mm depth) was placed directly over the skin surface (Grant *et al*., 2002; 2005). The probe was fixed in position over the skin with a normal laboratory manipulator by clamping onto a black acetal shank. Following baseline recording for 5 min, 20 μL of cinnamaldehyde (10%) was topically applied to the ipsilateral ear and 20 μL of vehicle (10% DMSO in ethanol) on the contralateral ear. Blood flow was subsequently measured for 30 min. Blood flow data were collected as flux units that are proportional to blood flow and expressed as area under the recorded flux versus time trace for the entire recording period following topical treatment (×10^3^ flux units). This model was first developed by Grant *et al*. (2002), and the simultaneous measurement of blood flow allows each mouse to serve as its own control, reducing animal use (Grant *et al*., 2002; 2005; Starr *et al*., 2008; Aubdool *et al*., 2014).

In some experiments, a full‐field perfusion imager (FLPI, Moor Instruments Ltd) was also used under a similar set‐up to assess blood flow on the whole ear skin (Starr *et al*., 2008; Pozsgai *et al*., 2010; Aubdool *et al*., 2014). The FLPI works on the same principle as the laser Doppler flowmeter's probe, except that it uses laser speckle imaging to produce high‐resolution imaging of real‐time assessment of skin blood flow prior to and following treatment, at regular 1 min intervals. Comparison studies examining the similarities and differences between both techniques have shown that there is a high correlation (*R* ^2^ = 0.98) in cerebral blood flow measurements in adjacent areas (Dunn *et al*., 2001). Blood flow traces were created by the image processing software MoorFLPI measurement V3.0 (Moor Instruments Ltd) at regular intervals of 4 s and analysed similarly to experiments using the laser Doppler flowmeter.

### Measurement of nitrite formation using the Griess assay

Nitrite (NO_2_^−^)/nitrate (NO_3_^−^) content was assessed by the Griess assay in vehicle‐ and cinnamaldehyde‐treated ear skin tissues at 30 min following treatment. Skin tissues were homogenized in acetonitrile, and homogenates were then centrifuged (8000 *g*, 4°C, 10 min) and supernatant was collected. NO_3_^−^ content was reduced to NO_2_^−^ by incubating 80 μL of sample with 20 μL of 1 U·mL^−1^ nitrate reductase and 10 μL of 1 mM NADPH for 30 min at 37°C (Fernandes *et al*., 2012). This was followed by the addition of 100 μL of Griess reagent (5% H_3_PO_4_ containing 1% sulfanilic acid and 0.1% *N*‐1‐napthylethylenediamine) (Fluka, Switzerland) and further incubated for another 30 min at 37°C. Absorbance was read at 550 nm using a spectrophotometer (SpectraMax 190, Molecular Devices Corporation, CA, USA). The absorbance of each sample was, after subtracting the background reading, compared against a standard curve (0–300 μM sodium nitrite) and expressed as NO^x^ levels mg^−1^ of tissue protein. All reagents were purchased from Sigma (UK).

### Superoxide measurements using the Lucigenin assay

Superoxide release was measured by chemiluminescence using bis‐*N*‐methylacridinium nitrate (Lucigenin, Sigma), as previously described (Fernandes *et al*., 2013; Aubdool *et al*., 2014). Following 30 min of topical treatment, vehicle‐ and cinnamaldehyde‐treated ear skin tissues were prepared, as described previously (Fernandes *et al*., 2013; Aubdool *et al*., 2014). The samples were placed in 100 μL of modified Krebs' buffer (composition of 131 mM NaCl, 5.6 mM KCl, 25 mM NaHCO_3_, 1 mM NaH_2_PO_4_·H_2_O, 5 mM glucose, 5 mM HEPES, 100 μM L‐arginine, 2.5 mM CaCl_2_, 1 mM MgCl_2_ and 100 μM NADPH, pH 7.4), and a further 100 μL of modified Krebs' buffer containing Lucigenin (10 mM) and NADPH (500 μM) was added to each sample with or without SOD (50 U·mL^−1^). Chemiluminescence was measured after 5 min using a GloMax 20/20 luminometer (Promega, UK). Results are expressed as the difference in the relative light units mg^−1^ of protein in the presence and absence of SOD after subtraction of background luminescence (Fernandes *et al*., 2013). All reagents were purchased from Sigma.

### Preparation of lysates and western blotting

Mice ear skin tissues were collected 30 min post‐treatment of vehicle and cinnamaldehyde, and snap frozen at −80°C until processing. Tissue was then lysed in SDS lysis buffer containing protease (1 tablet per 50 mL, Roche) and phosphatase (1 tablet per mL, Roche) inhibitor. Lysates were clarified by centrifuging at 2600 *g* for 10 min at 4°C. Protein concentration was assessed using the Bradford dye‐binding method kit (Bio‐Rad). Fifty micrograms of protein was loaded and separated by SDS‐PAGE and transferred to PVDF membranes using a semi‐dry technique (Aubdool *et al*., 2014). Membranes were blocked with 5% milk in PBS containing 0.1% Tween and incubated with primary antibodies against nitrotyrosine (1:500 dilution, Abcam ab61392) (Smillie *et al*., 2014) and loading control β‐actin (1:2000, Sigma A1978) dilution in 3% BSA in PBS and 0.1% Tween for 16 h at 4°C. Membranes were washed further with PBS 0.1% Tween and incubated with a horseradish peroxidase conjugated anti‐mouse secondary antibody (1:2000/5000 dilution, Sigma). Proteins were detected by enhanced chemiluminescence (Piercenet, UK) and densitometric analysis performed using Image J analysis software (NIH, USA). Total nitrotyrosine signal was calculated and normalized to the loading control β‐actin.

### Data analysis

We have adhered to the study design and analysis guidelines for British Journal of Pharmacology (Curtis *et al*. 2015). All data are presented as mean ± SEM. Most experiments in this study involved four groups and hence, a power analysis for a two‐way ANOVA design was used, based on previously published data from the mouse ear model (Grant *et al*., 2002; Starr *et al*., 2008; Pozsgai *et al*., 2010; Graepel *et al*., 2011; Aubdool *et al*., 2014). Sample size in this study varied between *n* = 4–7 (independent values per experiment). Statistical analysis was performed using GraphPad Prism (Version 5.0) software. Data statistical analysis was performed using Student's two‐tailed paired *t*‐test or two‐way ANOVA followed by Bonferroni's comparison *post hoc* test. Only a sample size ≥5 was subjected to statistical analysis as per the journal's guidelines. *P* < 0.05 represented a significant difference between groups.

### Materials

All drugs were purchased from Sigma unless otherwise stated. HC030031 (Tocris, UK) was dissolved in 10% DMSO in saline and administered i.p*.* at 100 mg·kg^−1^ (McNamara *et al*., 2007; Aubdool *et al*., 2014). Ruthenium red was dissolved in saline and administered i.p. at 3 mg·kg^−1^ (Cordova *et al*., 2011). CGRP_8–37_ was dissolved in 0.01% BSA and administered i.v. at 400 nmol·kg^−1^ (Tocris) (Grant *et al*., 2002; Aubdool *et al*., 2014). SR140333 (a gift from Dr X. Emonds‐Alt, Sanofi, Toulouse, France) was dissolved in saline and administered i.v. at 480 nmol·kg^−1^ (Starr *et al*., 2008; Aubdool *et al*., 2014). Glibenclamide was used i.v. at 20 mg·kg^−1^ (Buckingham *et al*., 1989). L‐NAME, SMTC or 1400 W was administered i.v*.* at 15 mg·kg^−1^ (Starr *et al*., 2008; Aubdool *et al*., 2014), 10 mg·kg^−1^ (Gozal *et al*., 1996; Aubdool *et al*., 2014) and 3 mg·kg^−1^ (Raimura *et al*., 2013) respectively. SMTC is 10‐fold more potent against nNOS than endothelial NOS (eNOS) (Furfine *et al*., 1994) and 1400 W is >50‐fold more potent against inducible NOS (iNOS) than eNOS (Garvey *et al*., 1997). Indomethacin was dissolved in 5% NaHCO_3_ in saline and administered i.v. at 20 mg·kg^−1^ and s.c*.* at 5 mg·kg^−1^ (Starr *et al*., 2008). *N*‐acetylcysteine and TEMPOL (Tocris) were administered i.p*.* at 300 mg·kg^−1^ (Zwingmann and Bilodeau, 2006) and i.v. at 30 mg·kg^−1^ (Starr *et al*., 2008; Aubdool *et al*., 2014) respectively. Catalase or its denatured form was administered i.p. at 25 000 U·kg^−1^ (Starr *et al*., 2008). Deferoxamine and apocynin were administered at 25 mg·kg^−1^ i.p*.* and 20 mg·kg^−1^ i.v. (Starr *et al*., 2008) respectively. 5,10,15,20‐Tetrakis(4‐sulfonatophenyl)porphyrinato iron (III), chloride (FeTPPS) or the iron‐free form of FeTPPS (TPPS, negative control) (Santa Cruz Biotechnology, Germany) were administered i.v. at a dose of 30 mg·kg^−1^ (Salvemini *et al*., 1998). These drugs were administered i.v. 5 min, i.p. 30 min and s.c. 60 min before baseline measurements. The doses used were chosen based on previous and preliminary studies.

## Results

### Cinnamaldehyde‐induced vasodilatation is dependent on TRPA1

**Figure 1:**
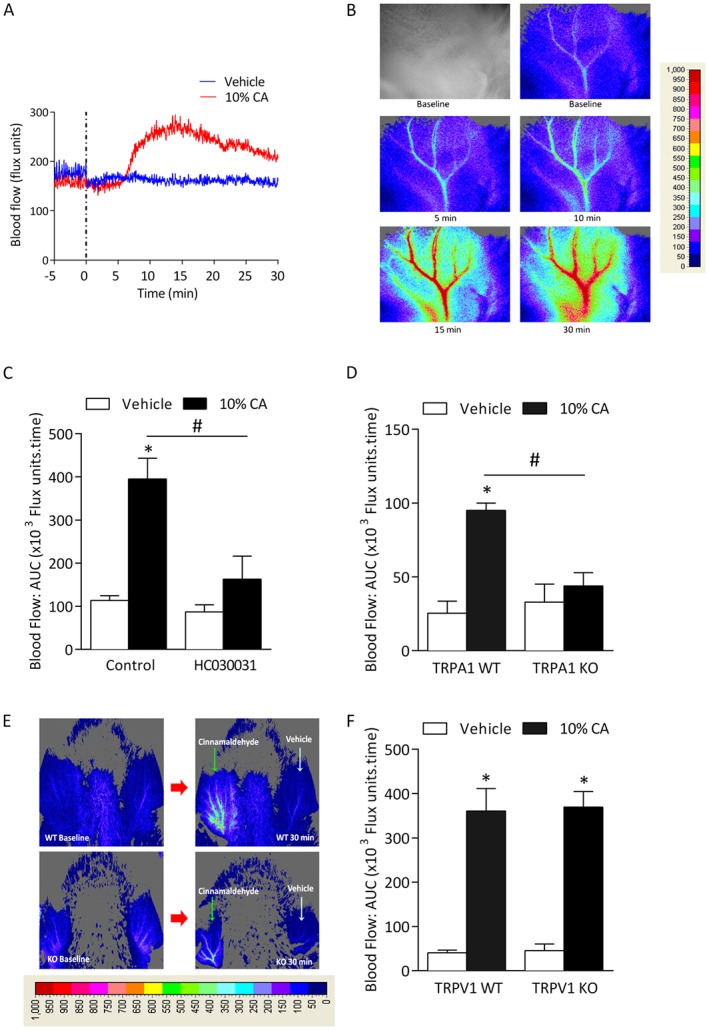
Cinnamaldehyde (CA)‐induced vasodilatation is dependent on TRPA1. Blood flow was measured in response to topical cinnamaldehyde (10% CA) and vehicle (10% DMSO in ethanol) in the anaesthetized mouse ear. Results recorded over 30 min and analysed as AUC. (A) Representative blood flow trace of CA‐induced response in WT mouse. Dotted lines represent topical administration of CA or vehicle. (B) Representative blood flow response as observed in pseudo‐colour images alongside grey/black image by laser speckle imager at baseline, 5‐30 min following treatment in WT mouse. (C) Group mean data for CA‐induced vasodilatation in WT mice pretreated with the TRPA1 antagonist HC030031 (100 mg·kg^−1^, *n* = 5) or control (10% DMSO in saline, *n* = 6). (D) Group mean data for CA‐induced vasodilatation in TRPA1 WT and KO mice (*n* = 5). (E) Representative blood flow response as observed in pseudo‐colour images by laser speckle imager at baseline and 30 min following treatment in TRPA1 WT and KO mouse. (F) Group mean data for CA‐induced vasodilatation in WT and TRPV1 KO mice (*n* = 5). All errors indicate SEM. *P < 0.05 versus vehicle‐treated ears of WT mice; #*P* < 0.05 versus CA‐treated ears of WT mice (2‐way ANOVA and Bonferroni *post hoc* test).

Topical application of cinnamaldehyde (1–30%) caused an increase in blood flow following treatment compared with vehicle‐treated ears in WT mice, which lasted for 30 min and returned to baseline by 60 min (Figure 1A and B; Supporting Information Fig. S1 and S2), as determined by laser Doppler flowmetry. The dose of 10% cinnamaldehyde was chosen for the subsequent studies as this dose mediates an axon reflex flare reaction on the forearm of healthy subjects, with an increase in blood flow (Namer *et al*., 2005), and here, it evoked a significant reproducible increase in skin blood flow (Supporting Information Fig. S1).

To determine the role of TRP channels in cinnamaldehyde‐induced vasodilatation, WT mice were pre‐treated with the non‐selective cation channel blocker ruthenium red (3 mg·kg^−1^) (Cordova *et al*., 2011), and a significant decrease in cinnamaldehyde‐induced vasodilatation was observed compared with control groups (Supporting Information Fig. S3). More specifically, the involvement of TRPA1 was investigated using the selective TRPA1 antagonist HC030031 (100 mg·kg^−1^) (McNamara *et al*., 2007; Aubdool *et al*., 2014) in WT mice. Figure 1C shows that cinnamaldehyde‐induced vasodilatation was abolished in HC030031 pre‐treated WT mice compared with control, supporting our previous finding (Aubdool *et al*., 2014). The role of TRPA1 is confirmed as cinnamaldehyde‐induced vasodilatation was absent in TRPA1 KO mice (Figure 1D and E).

As TRPA1 is co‐expressed in 60–75% of TRPV1‐expressing sensory neurons (Story *et al*., 2003; Kobayashi *et al*., 2005), we determined the selectivity of cinnamaldehyde by examining its effects on TRPV1 using WT and TRPV1 KO mice. The genetic deletion of TRPV1 had no significant effect on cinnamaldehyde‐induced vasodilatation (Figure 1F).

### Cinnamaldehyde‐induced vasodilatation is dependent on sensory neuropeptide CGRP

**Figure 2:**
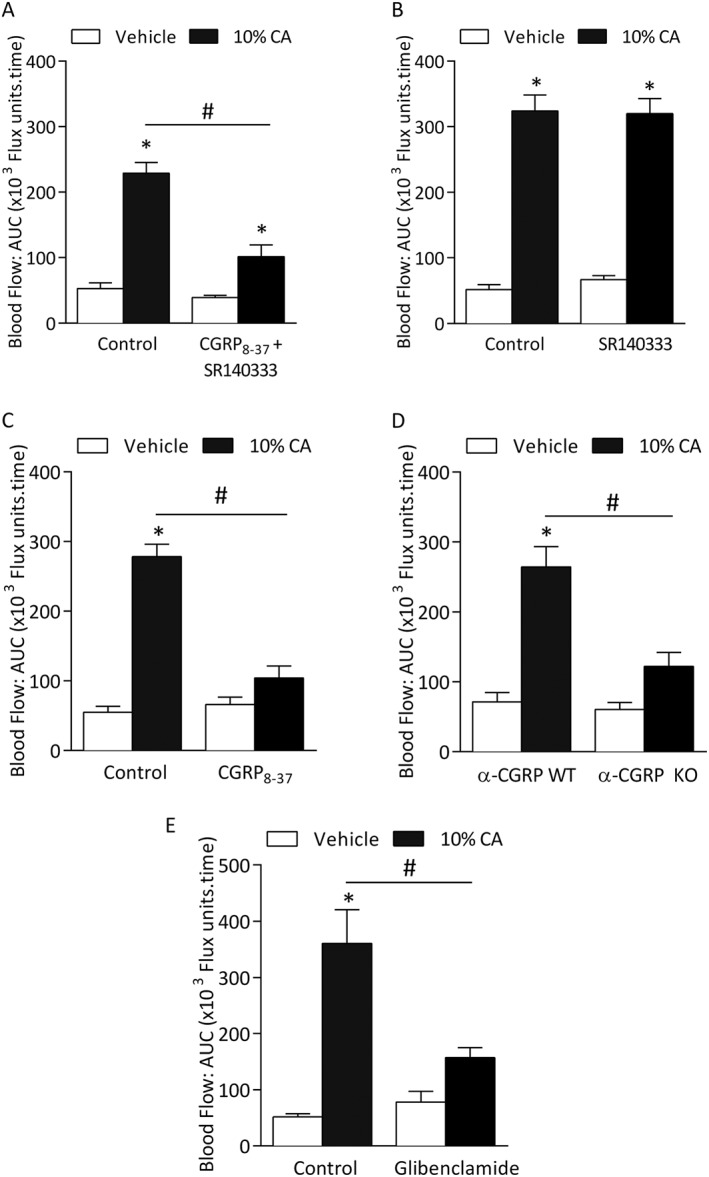
Cinnamaldehyde (CA)‐induced vasodilatation is dependent on CGRP. Blood flow was measured in response to topical cinnamaldehyde (10% CA) and vehicle (10% DMSO in ethanol) in the anaesthetized mouse ear. Results recorded over 30 min and analysed as AUC. Group mean data for CA‐induced vasodilatation in WT mice pretreated with (A) a combination of CGRP receptor antagonist CGRP_8‐37_ (400 nmol•kg^−1^) and substance P NK1 receptor antagonist SR140333 (480 nmol•kg^−1^, *n* = 5) or control (0.01% BSA in saline, n = 5), (B) SR140333 alone or control (saline, *n* = 5) and (C) CGRP_8‐37_ alone or control (0.01% BSA in saline, *n* = 5). (D) CA‐induced vasodilatation in α‐CGRP WT and KO mice (*n* = 5). Effects of pretreatment of (E) ATP‐sensitive K^+^ channels (KATP) blocker glibenclamide (20 mg•kg^−1^, *n* = 5). All errors indicate SEM. *P < 0.05 versus vehicle‐treated ears of WT mice; #*P* < 0.05 versus CA‐treated ears of WT mice (2‐way ANOVA and Bonferroni *post hoc* test).

The role of the two major vasodilator neuropeptides CGRP and substance P was investigated in cinnamaldehyde‐induced vasodilatation. Pre‐treatment with both neuropeptide receptor antagonists mixed together caused a significant decrease in cinnamaldehyde‐induced vasodilatation in WT mice (Figure 2A). Whilst pre‐treatment with SR140333 alone had no significant effect on this response (Figure 2B), WT mice pre‐treated with CGRP_8–37_ alone abolished cinnamaldehyde‐induced increase in blood flow responses (Figure 2C), indicating the major role of CGRP. In a complementary manner, a similar response was observed in α‐CGRP WT and KO mice (Figure 2D). Because CGRP‐induced vasodilatation is dependent on the activation of K_ATP_ channels, we investigated the effects of the K_ATP_ channel blocker glibenclamide (Buckingham *et al*., 1989). Pre‐treatment with glibenclamide caused a significant reduction in vasodilatation, supporting the importance of K_ATP_ channels in mediating this response (Figure 2E).

### nNOS‐derived NO and neuropeptides are involved in cinnamaldehyde‐induced vasodilatation

We subsequently investigated the role of other classical vasodilators such as prostaglandins and NO. WT mice pretreated with the cyclooxygenase inhibitor indomethacin (20 mg·kg^−1^) (Starr *et al*., 2008) showed no significant changes on cinnamaldehyde‐induced neurogenic vasodilatation (Supporting Information Fig. S3).

**Figure 3:**
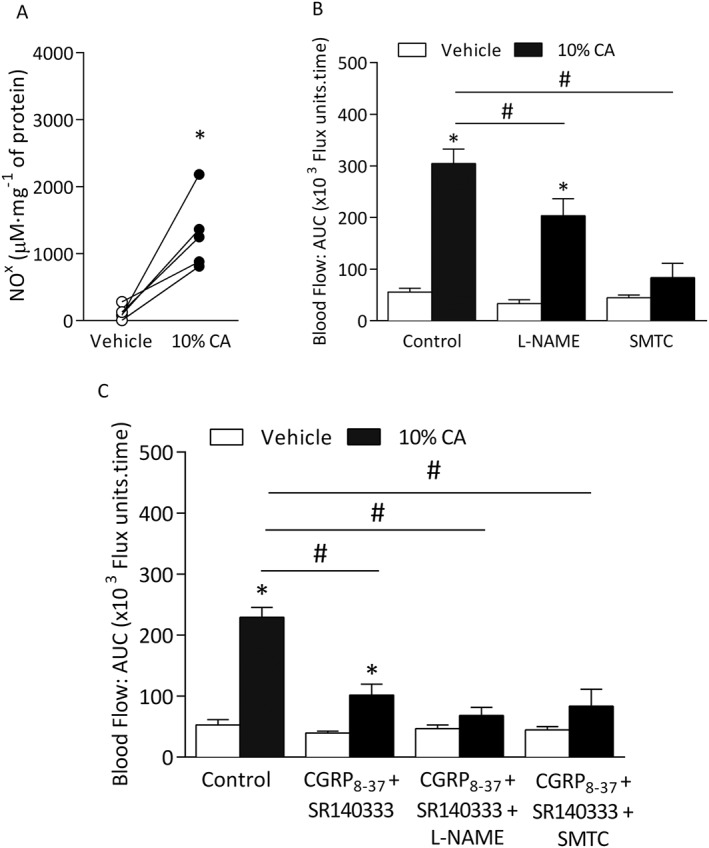
Cinnamaldehyde (CA)‐induced vasodilatation is dependent on nNOS‐derived NO. Blood flow was measured in response to topical cinnamaldehyde (10% CA) and vehicle (10% DMSO in ethanol) in the anaesthetized mouse ear. Results recorded over 30 min and analysed as AUC. (A) Nitrite (NO_2_^−^)/nitrate (NO_3_^−^) levels as shown indicating NO formation (NO^x^) using Griess assay in ear tissues of WT mice treated with vehicle or 10% CA for 30 min (*n* = 5). (B) Group mean data for CA‐induced vasodilatation in WT mice pretreated with the non‐selective NOS inhibitor L‐NAME (15 mg•kg^−1^, *n* = 6), nNOS inhibitor SMTC (10 mg•kg^−1^, *n* = 7) or control (saline, *n* = 6) alone. (C) Group mean data for CA‐induced vasodilatation in WT mice pretreated with control (saline, *n* = 5), a combination of CGRP_8‐37_ (400 nmol•kg^−1^) and SR140333 (480 nmol•kg^−1^) alone (*n* = 5) or with L‐NAME (*n* = 5) or SMTC (*n* = 6). All errors indicate SEM. *P < 0.05 versus vehicle‐treated ears of WT mice; #*P* < 0.05 versus CA‐treated ears of WT mice (2‐way ANOVA, Bonferroni *post hoc* test or Student's two‐tailed *t*‐test).

We next examined the formation of NO by assessing nitrite levels using the Griess assay. Ear tissues treated with cinnamaldehyde for 30 min showed an increase in NO formation compared with vehicle‐treated tissues (Figure 3A). Pre‐treatment with the non‐selective NOS inhibitor L‐NAME (15 mg·kg^−1^) (Starr *et al*., 2008) caused a significant reduction in cinnamaldehyde‐induced increase in blood flow compared with control (Figure 3B), but without significant effect on vehicle‐treated ear blood flow. Moreover, co‐treatment with L‐NAME, CGRP_8–37_ and SR140333 (Aubdool *et al*., 2014) abolished the cinnamaldehyde‐induced vasodilatation, comparable with the control group (Figure 3C and Table 1).

**Table 1:** Effects of neuropeptide receptor antagonists and NOS inhibitors on cinnamaldehyde‐induced vasodilatation

Treatment	*n*	Vehicle (20 μL)	CA (10%)
Control	6	55.7 ± 7.4	304.4 ± 28.1^*^
L‐NAME	5	33.2 ± 7.2	203.5± 44.6^*^ ^,^ ^#^
SMTC	6	44.6 ± 5.1	83.2 ± 27.9^*^ ^,^ ^#^
Control	5	52.8 ± 8.6	228.9 ± 16.3^*^
CGRP_8–37_ + SR140333	5	39.4 ± 3.1	101.5 ± 17.8^*^ ^,^ ^#^
CGRP_8–37_ + SR140333 + L‐NAME	5	46.7 ± 6.1	68.1 ± 13.3^#^
CGRP_8–37_ + SR140333 + SMTC	6	44.6 ± 5.1	83.2 ± 27.9^#^

Blood flow was measured in response to topical cinnamaldehyde (10% CA) and vehicle (10% DMSO in ethanol) in the anaesthetized mouse ear. Results were recorded over 30 min and analysed as AUC. WT mice were pre‐treated with the non‐selective NOS inhibitor L‐NAME (15 mg·kg^−1^, *n* = 6), nNOS inhibitor SMTC (10 mg·kg^−1^, *n* = 7) or control (saline, *n* = 6) with and without CGRP_8–37_ (400 nmol·kg^−1^) and SR140333 (480 nmol·kg^−1^) (*n* = 5–6). All errors indicate SEM.

* *P* < 0.05 versus vehicle‐treated ears of WT mice.

# *P* < 0.05 versus CA‐treated ears of WT mice (2‐way ANOVA and Bonferroni *post hoc* test).

To determine the source of NO, we used iNOS‐specific and nNOS‐specific inhibitors. Whilst the selective iNOS inhibitor 1400 W (3 mg·kg^−1^) (Raimura *et al*., 2013) with or without the neuropeptide receptor antagonists had no further significant reduction in blood flow (Supporting Information Fig. S4), the nNOS inhibitor (SMTC) (Gozal *et al*., 1996; Aubdool *et al*., 2014) inhibited this response (Figure 3B), and co‐treatment with neuropeptide receptor antagonists further attenuated this response (Figure 3C). These results highlight the major role of nNOS in cinnamaldehyde‐induced vasodilatation.

### A role for superoxide and peroxynitrite in cinnamaldehyde‐induced vasodilatation

**Figure 4:**
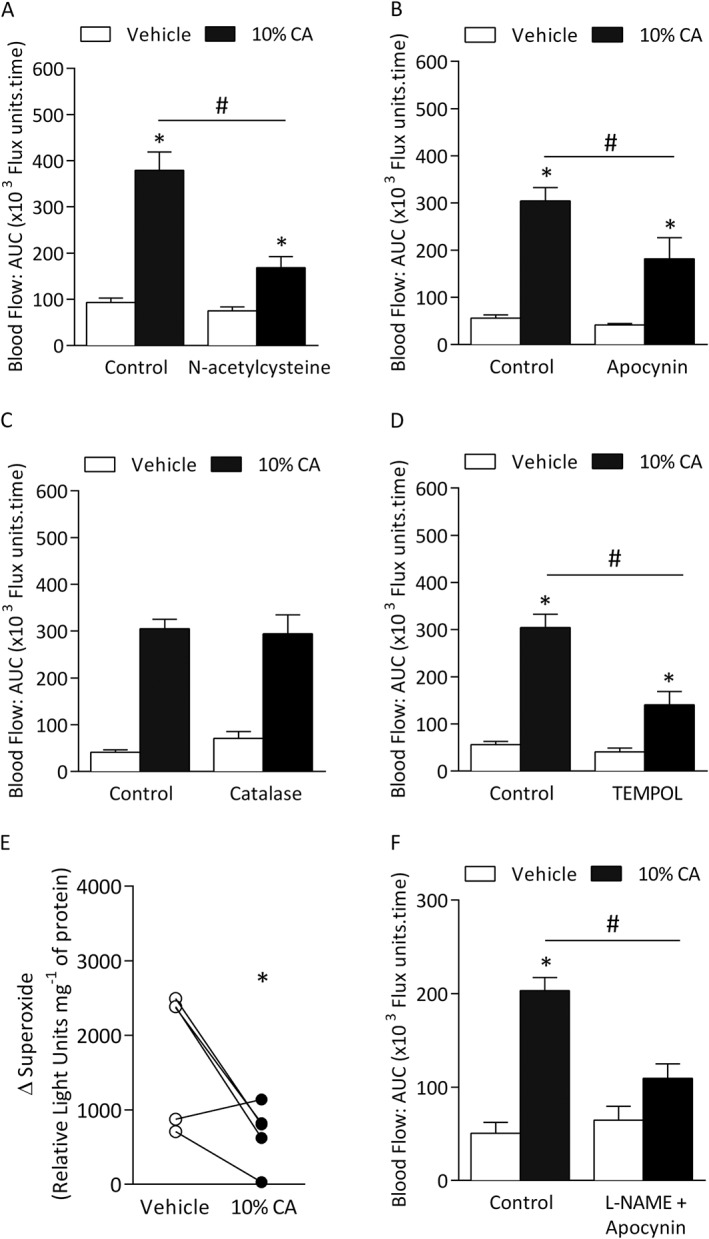
A role for ROS in mediating cinnamaldehyde (CA)‐induced vasodilatation. Blood flow was measured in response to topical cinnamaldehyde (10% CA) and vehicle (10% DMSO in ethanol) in the anaesthetized mouse ear. Results recorded over 30 min and analysed as AUC. Group mean data for CA‐induced vasodilatation in WT mice pretreated with the (A) ROS scavenger N‐acetylcysteine (300 mg•kg^−1^, *n* = 6) or control (saline, *n* = 6), (B) apocynin (20 mg•kg^−1^, *n* = 7) or control (saline, *n* = 6), (C) H2O2 scavenger catalase (*n* = 4) or control (denatured catalase enzyme, *n* = 4) and (D) SOD mimetic TEMPOL (30 mg•kg^−1^, *n* = 5) or control (saline, *n* = 5). (E) Superoxide levels in vehicle‐treated or CA‐treated ears of WT mice at 30 min following treatment, as measured by Lucigenin assay (*n* = 5). (F) Group mean data for CA‐induced vasodilatation in WT mice pre‐treated L‐NAME and apocynin or control (saline, *n* = 6). Data represent mean ± SEM. *P < 0.05 versus vehicle‐treated ears of WT mice; #*P* < 0.05 versus CA‐treated ears of WT mice (2‐way ANOVA, Bonferroni *post hoc* test or Student's two‐tailed *t*‐test).

ROS are involved in modulating neurogenic‐induced vasodilatation following sensory neuron activation by capsaicin in the mouse ear (Starr *et al*., 2008). Pre‐treatment with the antioxidant N‐acetylcysteine (300 mg·kg^−1^) (Zwingmann and Bilodeau, 2006) or apocynin (acting as a ROS scavenger at 20 mg·kg^−1^) (Heumuller *et al*., 2008) caused a significant decrease in cinnamaldehyde‐induced vasodilatation compared with control group (Figure 4A and B). To investigate the role of hydrogen peroxide (H_2_O_2_), the H_2_O_2_ scavenger catalase (25 000 U·kg^−1^) (Starr *et al*., 2008) was used. Pre‐treatment with catalase had no effect on cinnamaldehyde‐induced responses compared with WT mice treated with the deactivated enzyme catalase (control, Figure 4C). Using the membrane permeable SOD mimetic TEMPOL (30 mg·kg^−1^) (Starr *et al*., 2008), we observed reduced vasodilator response compared with control group in WT mice (Figure 4D). Treatment with cinnamaldehyde resulted in reduced superoxide level in the ear tissue samples, suggesting that superoxide may react with another mediator (possibly with the increase in NO; Figure 3A) forming a product, which contributes to cinnamaldehyde‐induced vasodilatation (Figure 4E). As co‐treatment using a NOS inhibitor (L‐NAME) and antioxidant apocynin showed reduction in cinnamaldehyde‐induced vasodilatation, it is possible that superoxide reacts with NO to produce RONS such as peroxynitrite (Figure 4F).

**Figure 5:**
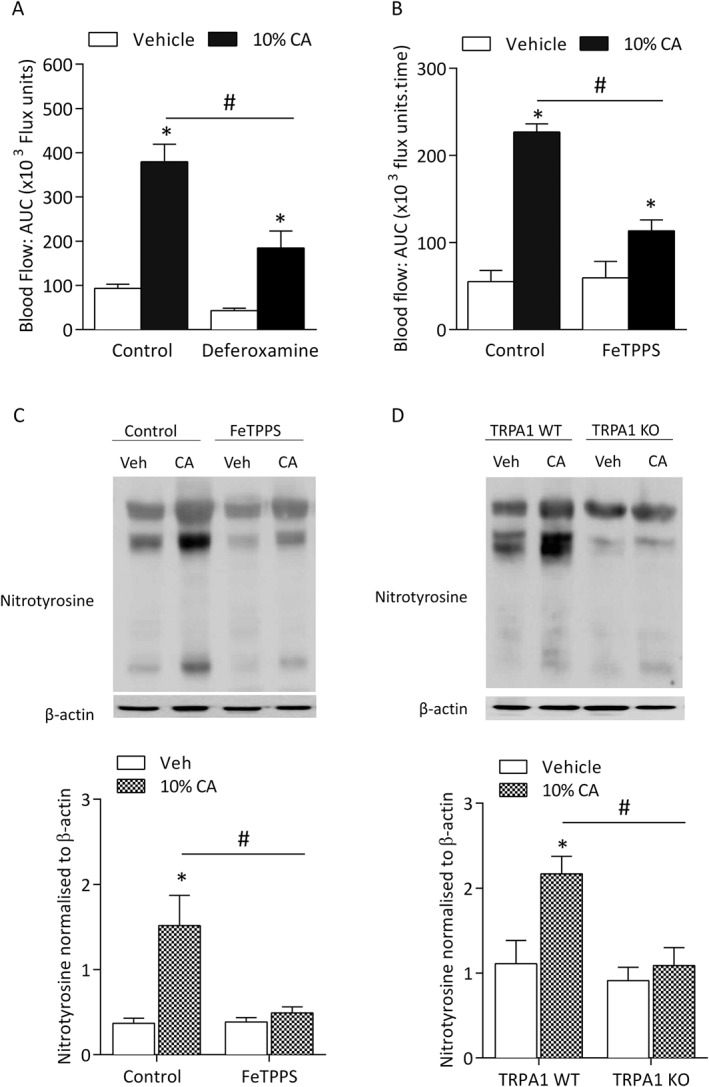
Peroxynitrite (ONOO^−^) is involved in cinnamaldehyde (CA)‐induced vasodilatation. Blood flow was measured in response to topical cinnamaldehyde (10% CA) and vehicle (10% DMSO in ethanol) in the anaesthetized mouse ear. Results recorded over 30 min and analysed as AUC. Group mean data for CA‐induced vasodilatation in WT mice pretreated with (A) deferoxamine (25 mg•kg^−1^, *n* = 6) or control (saline, *n* = 6) or (B) the peroxynitrite scavenger FeTPPs (30 mg•kg^−1^, *n* = 5) or control (*n* = 5). (C) Representative western blot (top panel) and densitometric analysis (bottom panel, *n* = 5) of nitrotyrosine protein expression, as a marker of peroxynitrite formation and β‐actin (loading control) in vehicle‐treated and CA‐treated ear tissues of WT mice pretreated with FeTPPs or control at 30 min following treatment (left panel). (D) Representative western blot (top panel) and densitometric analysis (bottom panel, *n* = 5) of nitrotyrosine protein expression and β‐actin (loading control) in vehicle‐treated and CA‐treated ear tissues of TRPA1 WT and KO mice at 30 min following treatment (left panel). Data represent mean ± SEM. *P < 0.05 versus vehicle‐treated ears of WT mice; #*P* < 0.05 versus CA‐treated ears of WT mice (2‐way ANOVA and Bonferroni *post hoc* test).

To further investigate the role of peroxynitrite, the iron chelator and peroxynitrite scavenger deferoxamine (25 mg·kg^−1^) (Beckman *et al*., 1990) or the selective peroxynitrite scavenger FeTPPS (30 mg·kg^−1^) was administered in WT mice and caused a significant reduction in cinnamaldehyde‐induced vasodilatation (Figure 5A and B). There was no change in responses in WT mice pre‐treated with the iron‐free form of FeTPPS, TPPS (negative control; Supporting Information Fig. S5). The efficacy of FeTPPS to scavenge peroxynitrite was further confirmed by western blotting to detect protein tyrosine nitration using an antibody against nitrotyrosine residues (Figure 5C and D and Supporting Information Fig. S6). Cinnamaldehyde induced an increase in nitrosylated proteins, which was significantly reduced in WT mice pre‐treated with FeTPPS (Figure 5C) and in TRPA1 KO mice (Figure 5D), suggesting a potential role for peroxynitrite downstream of cinnamaldehyde activation of TRPA1 in mediating the observed vasodilatation.

## Discussion

This study shows that TRPA1 stimulation by cinnamaldehyde can mediate neurogenic vasodilatation in the peripheral vasculature mediated to a large extent by the sensory neuropeptide CGRP. Cinnamaldehyde‐induced neurogenic vasodilatation was (i) absent in TRPA1 KO mice, (ii) blocked by co‐treatment with NOS inhibitor and antioxidant and (iii) resulted in increased protein tyrosine nitration, which was inhibited by the peroxynitrite scavenger FeTPPS and absent in TRPA1 KO mice, implying that RONS production is an essential component of TRPA1‐mediated neurogenic vasodilatation. We show for the first time that independent of TRPV1, RONS are involved in cinnamaldehyde‐mediated vasodilatation, possibly generated through a reaction between NO and superoxide downstream of TRPA1 activation.

Studies investigating the mechanisms underlying TRPA1 activation have relied on selective agonists, and to date, cinnamaldehyde has proven to be among the most selective chemical agonist of TRPA1 *in vitro* (Bodkin and Brain, 2011). Cinnamaldehyde excites a subset of sensory neurons highly enriched in cold‐sensitive neurons *in vitro* (Bandell *et al*., 2004), and at a dose of 10%, it mediates an axon reflex flare reaction on the forearm of healthy subjects (Namer *et al*., 2005). However, it remains unknown whether this response is TRPA1‐dependent in humans. Following our previous evidence that cinnamaldehyde increases blood flow in the hindpaw vasculature and influences changes in blood pressure *in vivo* via TRPA1 activation (Pozsgai *et al*., 2010), we provide evidence of its vasodilator effects in the mouse ear vasculature, in a TRPA1‐dependent manner. Despite suggestions of possible interactions between TRPA1 and TRPV1 on sensory neurons (Kobayashi *et al*., 2005), cinnamaldehyde‐induced vasodilatation was not affected by TRPV1 gene deletion. We also demonstrated that the pharmacological blockade of transient receptor potential melastatin‐8 using AMTB did not affect this response (Aubdool *et al*., 2014). These combined results imply that TRPA1 activation can act alone to mediate cinnamaldehyde‐evoked vasodilatation in the skin.

TRPA1‐dependent vasodilatation has been previously shown to be mediated by sensory neuropeptides (Grant *et al*., 2005; Pozsgai *et al*., 2010; Graepel *et al*., 2011; Aubdool *et al*., 2014). Pharmacological blockade of the neurokinin‐1 (NK_1_) receptor using SR140333 alone, at doses previously shown to inhibit substance P‐induced vasodilatation (Grant *et al*., 2002; Starr *et al*., 2008) did not affect TRPA1‐induced responses, suggesting that substance P is not primarily involved in this response, despite its established co‐localization with CGRP in sensory nerves. We have previously shown that both capsaicin‐ and mustard oil‐induced vasodilatation are not reduced in NK_1_ receptor knockout mice but blocked when these KO mice are pre‐treated with the CGRP receptor antagonist (Grant *et al*., 2002, 2005). This suggests that the NK_1_ receptor antagonist is only able to reduce vasodilatation once the CGRP receptor component has been removed.

**Figure 6:**
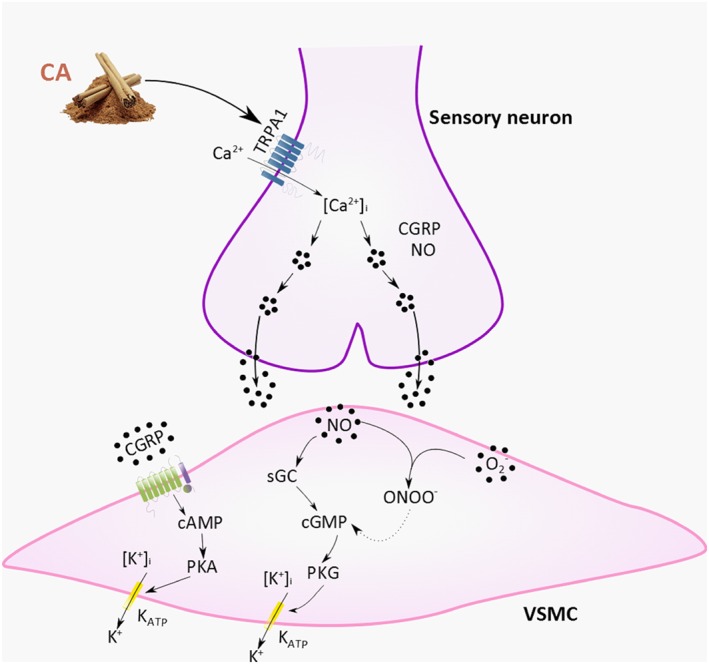
Schematic diagram of TRPA1‐induced vasodilatation involving CGRP, NO and peroxynitrite. Cinnamaldehyde activates TRPA1 on sensory neuron to mediate increase in [Ca^2+^], leading to the release of the neuropeptide CGRP and nNOS‐derived NO. CGRP acts on the CGRP receptor complex to increase cAMP, further phosphorylating PKA and opening KATP channels, mediating relaxation. NO acts on stimulating sGC to subsequently form cGMP, activating PKG, which causes reuptake of Ca^2+^ and opens K^+^ channels, leading to hyperpolarization of the membrane and further relaxing the VSMC. RONS, such as superoxide possibly being produced following TRPA1 activation, can react with NO to form ONOO^•^, maintaining VSMC relaxation. Abbreviations: CA, cinnamaldehyde; nNOS, neuronal NOS; ONOO^•^, peroxynitrite; O_2_^•^, superoxide; sGC, soluble guanylyl cyclase; K_ATP_, ATP‐sensitive K^+^ channels, VSMC, vascular smooth muscle cell. Dotted line represents proposed pathway.

Although α‐CGRP is a potent microvascular vasodilator in the cutaneous, coronary and cerebral vasculature, CGRP receptor antagonists do not affect baseline cardiovascular parameters in humans (Olesen *et al*., 2004) or rodents (Escott and Brain, 1993; Russell *et al*., 2014). Here, cinnamaldehyde‐induced vasodilatation was inhibited when the α‐CGRP gene was deleted or its receptors were blocked, consistent with previous findings using other TRPA1 activators in the vasculature (Pozsgai *et al*., 2012; Aubdool *et al*., 2014; Eberhardt *et al*., 2014). CGRP binds to its receptor complex and can mediate its vasodilator effects in a cAMP‐dependent pathway, mediating the opening of K_ATP_‐sensitive K^+^ channels (Nelson *et al*., 1990) (Figure 6). Indeed, pre‐treatment with glibenclamide significantly reduced cinnamaldehyde‐induced vasodilatation, suggesting that cinnamaldehyde may activate TRPA1 and release CGRP from sensory neurons, which further mediates vasodilatation by opening K_ATP_ channels on the vascular smooth muscle cells (Figure 6).

An interesting finding in this study was that whilst CGRP_8–37_ and not SR140333 was able to inhibit cinnamaldehyde‐induced vasodilatation, the simultaneous pharmacological blockade of both the CGRP and substance P NK_1_ receptors substantially reduced cinnamaldehyde‐induced vasodilatation. However, a small but significant vasodilatation remained, and this suggests a possible interaction between these neuropeptides, which have previously been reported (Grant *et al.*, 2005). Although the nature of the mechanism of this residual response remains to be determined, we hypothesize that the mediator involve may be NO. Stimulation of sensory nerves can also release NO to control vasoactive responses. Here, we found an increase in NO formation in cinnamaldehyde‐treated ear tissues, as measured by the Griess assay, and pre‐treatment with L‐NAME showed a marked decrease in cinnamaldehyde‐induced vasodilatation response. There are no selective eNOS isoform inhibitors available; however, a selective nNOS inhibitor SMTC blocked cinnamaldehyde‐induced vasodilatation. SMTC was used at a dose known to selectively target nNOS *in vivo*, with only transient increase in blood pressure in studies using conscious rats (Gozal *et al*., 1996). This finding builds on our recent discovery of a link between TRPA1 activation and nNOS‐derived NO (Aubdool *et al*., 2014). Pretreatment with L‐NAME or SMTC with the sensory neuropeptide receptor antagonists abolished cinnamaldehyde‐induced vasodilatation. Notably, NO has been previously reported to regulate release of CGRP but not the action of sensory vasodilator CGRP (Holzer and Jocic, 1994; Hughes and Brain, 1994; Kajekar *et al*., 1995; Bellamy *et al*., 2006). This is evident in studies where L‐NAME has no effect in reducing CGRP‐induced vasodilatation in the rabbit (Hughes and Brain, 1994) and rat (Ralevic *et al*., 1992) skin. Because both the CGRP receptor antagonist and nNOS inhibitor inhibit the cinnamaldehyde‐induced vasodilatation, this suggests that both mediators are involved in mediating TRPA1‐induced neurogenic vasodilatation, but possibly sequentially such that they are both able to influence the response at different stages (Figure 6).

It is evident from this study that the sensory neurons play a major role to elicit neurogenic‐dependent vasodilatation following TRPA1 activation by cinnamaldehyde in the skin. However, a vascular role of TRPA1 has been suggested. Whilst it is established that TRPA1 channels are present on the endothelium of cerebral arteries, the TRPA1 channel was not found in other vascular beds such as coronary, mesenteric or renal arterial endothelium in rats (Sullivan *et al*., 2015). It is presently unknown whether TRPA1 is present in the vascular beds of the mouse ear, and this requires further investigation, which is beyond the scope of this study. It has also been suggested that TRPA1 is present in keratinocytes (Kwan *et al.*, 2009), but the functional importance of this in vasodilatation is currently unclear. We hence propose that cinnamaldehyde acts on functional TRPA1 in the mouse ear skin to mediate neurogenic vasodilatation. Hydrogen sulphide (H_2_S) and nitroxyl (HNO) has recently been suggested as potential endogenous TRPA1 agonists of neurogenic vasodilatation (Pozsgai *et al*., 2012; Eberhardt *et al.*, 2014; Dux *et al.*, 2016).

CGRP‐ and substance P‐induced vasodilatation involves the participation of ROS (Starr *et al*., 2008). We showed that pretreatment with the powerful antioxidant *N*‐acetylcysteine significantly decreased this response. This is consistent with previous *in vitro* findings whereby *N*‐acetylcysteine blocked cinnamaldehyde‐induced ROS production in human leukaemia promyeolocytic cells (Ka *et al*., 2003). However, there is evidence that RONS such as peroxynitrite can react vigorously with thiol groups, oxidizing *N*‐acetylcysteine (Szabo *et al*., 2007) or possibly cinnamaldehyde, further interfering with its ability to activate TRPA1. To further confirm any potential role of ROS/RONS in our response, we investigated other ROS/RONS pathways modulators. Treatment with apocynin at a dose where it acts as a ROS scavenger rather than a selective NADPH oxidase (NOX) inhibitor (Heumuller *et al*., 2008) reduced cinnamaldehyde‐induced vasodilatation, further implying a novel role of RONS in this response.

CGRP‐ and substance P‐induced vasodilatation is inhibited by SOD and catalase, suggesting an involvement of superoxide and H_2_O_2_ (Starr *et al*., 2008). Here, whilst treatment with the H_2_O_2_ scavenger catalase had no effect on cinnamaldehyde‐induced vasodilatation, pre‐treatment with the SOD mimetic TEMPOL reduced cinnamaldehyde‐induced responses. This finding supports the partial involvement of SOD in the TRPA1‐mediated vascular responses (Aubdool *et al*., 2014). Furthermore, a significant decrease in superoxide levels was detected in cinnamaldehyde‐treated ear tissue samples, which can possibly result due to generation of RONS, peroxynitrite through an interaction between NO and superoxide. Indeed, co‐treatment of L‐NAME with apocynin blocked cinnamaldehyde‐induced vasodilatation, suggesting an important role for NO and ROS in mediating this vasodilator response.

Notably, we observed a significant increase in protein tyrosine nitration levels, as assessed by immunoblotting using a marker for peroxynitrite in cinnamaldehyde‐treated tissues. Cinnamaldehyde‐induced vasodilatation and associated increase in peroxynitrite formation was significant reduced in mice pre‐treated with the peroxynitrite scavenger FeTPPS. This provides strong evidence of a role of peroxynitrite in cinnamaldehyde‐induced vasodilatation. Peroxynitrite is known to exert vasorelaxation on peripheral blood vessels under physiological conditions (Beckman *et al*., 1990; Dowell and Martin, 1997) via stimulation of cGMP (Casey *et al*., 2012) and activation of K_ATP_ channels (Villa *et al*., 1994).

We further examined whether peroxynitrite is formed upstream or downstream of TRPA1 activation by cinnamaldehyde and demonstrated that cinnamaldehyde‐induced increase in protein tyrosine nitration was abolished in TRPA1 KO mice. Collectively, our results suggest that cinnamaldehyde‐induced RONS generation, especially peroxynitrite, is possibly involved downstream of TRPA1 activation by cinnamaldehyde (Figure 6B). However, once generated, we cannot exclude the possibility of RONS acting directly on TRPA1 to mediate vascular effects as TRPA1 can act as a ROS sensor (Andersson *et al*., 2008; Eberhardt *et al*., 2014; Andersson *et al*., 2015; Sullivan *et al*., 2015). Indeed, peroxynitrite can activate TRPA1 by oxidizing its cysteine residues (Andersson *et al*., 2015). There is also evidence showing that NADPH oxidase 2‐derived superoxide is converted to H_2_O_2_ and later to OH, leading to peroxidation of membrane lipids, which activates TRPA1 and causes dilatation in cerebral arteries (Sullivan *et al*., 2015). Whilst we show that H_2_O_2_ is not involved here, it is unknown whether cinnamaldehyde induces lipid peroxidation in our current study. Thus, it is possible that TRPA1‐induced peroxynitrite formation following cinnamaldehyde treatment can directly activate TRPA1, leading to a positive modulatory action of RONS on TRPA1 to mediate vasodilatation in the skin vasculature.

## Conclusion

In conclusion, our findings show that cinnamaldehyde can activate TRPA1 and emphasize the established important role of CGRP in mediating neurogenic vasodilatation in the microvasculature. We found that nNOS‐derived NO is important in mediating this response. We provide novel evidence for the regulatory role of RONS in TRPA1‐mediated neurogenic vasodilatation and demonstrated that peroxynitrite is generated downstream of TRPA1 activation by cinnamaldehyde. These results demonstrate the intimate links between neurogenic vasodilatation and oxidative mechanisms. Thus, we propose that TRPA1 plays a role in regulating vascular tone in the periphery and this mechanistic pathway may help in further understanding the role of TRPA1 in pathophysiological situations.

## Author contributions

A.A.A., X.K., N.A.‐K. and E.S.F. performed and analysed experiments. R.H. contributed to essential materials and interpretation of the superoxide assays. S.B. provided the TRPA1 KO mice. S.D.B. designed and managed the research study. A.A.A. and S.D.B. wrote the manuscript. All authors contributed to manuscript preparation.

## Conflict of interest

The authors declare no conflicts of interest.

## Declaration of transparency and scientific rigour

This Declaration acknowledges that this paper adheres to the principles for transparent reporting and scientific rigour of preclinical research recommended by funding agencies, publishers and other organisations engaged with supporting research.

## Supporting information


**Figure S1** Dose–response curve for cinnamaldehyde (CA)‐induced blood flow responses. Blood flow was measured in response to topical application of 20 μl of cinnamaldehyde (1‐30%) and vehicle (10% DMSO in ethanol) in the anaesthetised WT mice ear. Results were recorded over 30 min and analysed as area under the curve (AUC). All errors indicate SEM. (*n* = 5). *P < 0.05 vs. vehicle‐treated (two‐tailed Student's t‐test).
**Figure S2** Effect of 10% cinnamaldehyde (CA)‐induced blood flow responses. Blood flow was measured in response to topical application of 20 μl of cinnamaldehyde (10%) and vehicle (10% DMSO in ethanol) in the anaesthetised WT mice ear. (A) Representative blood flow trace of CA‐induced response in WT mouse. Dotted line represent topical administration of CA or vehicle. (B) Group mean data for CA‐induced vasodilatation in WT mice for blood flow responses recorded over 60 min and analysed as area under the curve (AUC). All errors indicate SEM (*n* = 4).
**Figure S3** Effects of inhibitors on cinnamaldehyde‐induced vasodilatation. Blood flow was measured in response to topical cinnamaldehyde (10% CA) and vehicle (10% DMSO in ethanol) in the anaesthetised mouse ear. Results recorded over 30 min and analysed as area under the curve (AUC). (A) WT mice were pre‐treated with the non‐selective cation channel blocker ruthenium red (3 mg kg‐1) or control (saline). (B) WT mice were pre‐treated with the non‐selective cyclooxygenase inhibitor indomethacin (20 mg kg‐1) or control (5% or 0.05% NaHCO3 in saline). Data shows mean + SEM. *P < 0.05 vs. vehicle‐treated, #*P* < 0.05 vs. CAtreated ears of WT mice (2‐WAY ANOVA, Bonferroni *post hoc* test).
**Figure S4** Cinnamaldehyde (CA)‐induced vasodilatation is not dependent on iNOS derived nitric oxide. Blood flow was measured in response to topical application of 20 μl of cinnamaldehyde (10% CA) and vehicle (10% DMSO in ethanol) in the anaesthetised mouse ear. Results were recorded over 30 min and analysed as area under the curve (AUC). Group mean data for CA‐induced vasodilatation in WT mice pre‐treated with (A) the selective iNOS inhibitor 1400 W alone (3 mg kg‐1, *n* = 9) or control (saline, *n* = 9) and (B) a combination of 1400 W (3 mg kg‐1) with CGRP8‐37 (400 nmol kg‐1) and SR140333 (480 nmol kg‐1) or control (saline, *n* = 5‐7). All errors indicate SEM. *P < 0.05 vs. vehicle‐treated, #*P* < 0.05 vs. CA‐treated ears of WT mice (2‐WAY ANOVA, Bonferroni post *hoc* test).
**Figure S5** Effects of Tetraphenylporphinesulfonate (TPPS) on cinnamaldehyde (CA)‐induced vasodilatation. Blood flow was measured in response to topical application of 20 μl of cinnamaldehyde (10% CA) and vehicle (10% DMSO in ethanol) in the anaesthetised mouse ear. Results were recorded over 30 min and analysed as area under the curve (AUC). Group mean data for CA‐induced vasodilatation in WT mice pre‐treated with TPPS (30 mg kg‐1, *n* = 5) or control (saline, *n* = 5). All errors indicate SEM. *P < 0.05 vs. vehicle‐treated ears of WT mice (2‐WAY ANOVA, Bonferroni *post hoc* test).
**Figure S6** Uncropped immunoblots for Figure 5C‐D displayed in the main figures. Immunoblots are developed using Syngene gel doc digital dark room system. A digital image of the membrane is acquired, following which, the immunoblot is developed to reveal the probed protein bands (kDa). (A) A merged image of the captured membrane and developed nitrotyrosine immunoblot (left panel), and uncropped immunoblot for nitrotyrosine for Figure 5C (right panel). (B) A merged image of the captured membrane and developed β‐actin immunoblot (left panel), and uncropped immunoblot for β‐actin for Figure 5C (right panel) for vehicle and cinnamaldehyde‐treated tissue samples in WT mice pre‐treated with FeTPPS (30 mg kg‐1) or control. (C) A merged image of the captured membrane and developed nitrotyrosine immunoblot (left panel), and uncropped immunoblot for nitrotyrosine for Figure 5D (right panel). (D) A merged image of the captured membrane and developed β‐actin immunoblot (left panel), and uncropped immunoblot for β‐actin for Figure 5D (right panel) for vehicle and cinnamaldehyde‐treated tissue samples in TRPA1 WT and KO mice. Boxed areas indicate the cropped regions displayed in Figure 5C‐D.

Supporting info item
