# Supplementary material for: TRPA1 activation leads to neurogenic vasodilatation: involvement of reactive oxygen nitrogen species in addition to CGRP and NO
Source: Br J Pharmacol. 2016 Jun 21;173(15):2419–33. doi: 10.1111/bph.13519 (PMC4945766; doi:10.1111/bph.13519)
Supplement: Supplementary file 1 — Supporting info item [file BPH-173-2419-s001.pdf]

Supporting Information for Online Publication Only

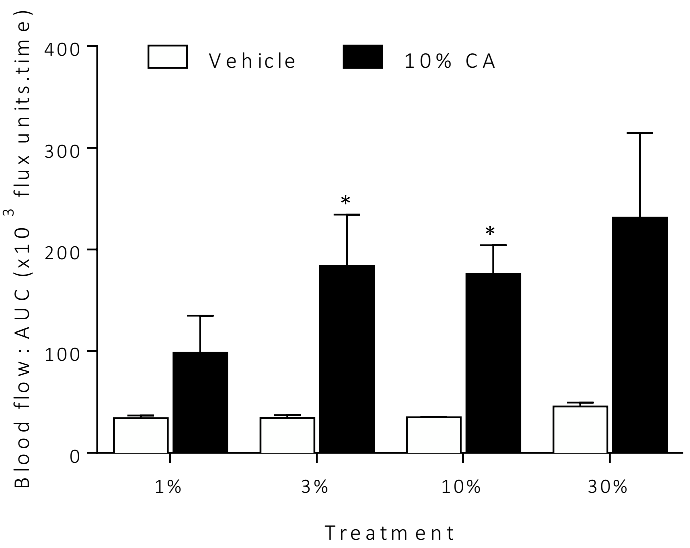

Figure S1 Dose-response curve for cinnamaldehyde (CA)-induced blood flow responses. Blood flow was measured in response to topical application of 20µl of cinnamaldehyde (1-30%) and vehicle (10% DMSO in ethanol) in the anaesthetised WT mice ear. Results were recorded over 30 min and analysed as area under the curve (AUC). All errors indicate SEM. (n=5). *p<0.05 vs vehicle-treated (two-tailed Student's t-test).

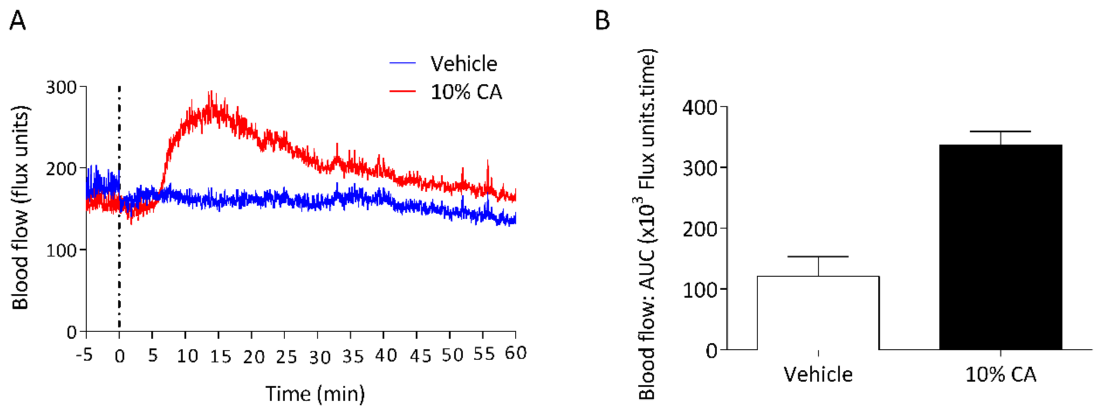

Figure S2 Effect of 10% cinnamaldehyde (CA)-induced blood flow responses. Blood flow was measured in response to topical application of 20μl of cinnamaldehyde (10%) and vehicle (10% DMSO in ethanol) in the anaesthetised WT mice ear. (***A***) Representative blood flow trace of CA-induced response in WT mouse. Dotted line represent topical administration of CA or vehicle. (***B***) Group mean data for CA-induced vasodilatation in WT mice for blood flow responses recorded over 60 min and analysed as area under the curve (AUC). All errors indicate SEM (n=4).

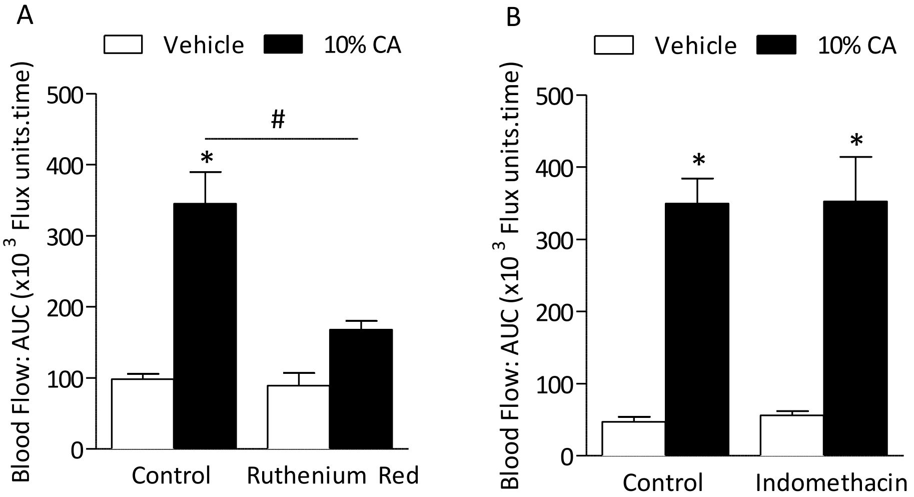

Figure S3 Effects of inhibitors on cinnamaldehyde-induced vasodilatation. Blood flow was measured in response to topical cinnamaldehyde (10% CA) and vehicle (10% DMSO in ethanol) in the anaesthetised mouse ear. Results recorded over 30 min and analysed as area under the curve (AUC). ***(A)*** WT mice were pre-treated with the non-selective cation channel blocker ruthenium red (3mg kg^{-1}) or control (saline). ***(B)*** WT mice were pre-treated with the non-selective cyclooxygenase inhibitor indomethacin (20mg kg^{-1}) or control (5% or 0.05% $NaHCO_3$ in saline). Data shows mean $\pm$ SEM. *$p<0.05$ vs vehicle-treated, #$p<0.05$ vs CA-treated ears of WT mice (2-WAY ANOVA, Bonferroni *post hoc* test).

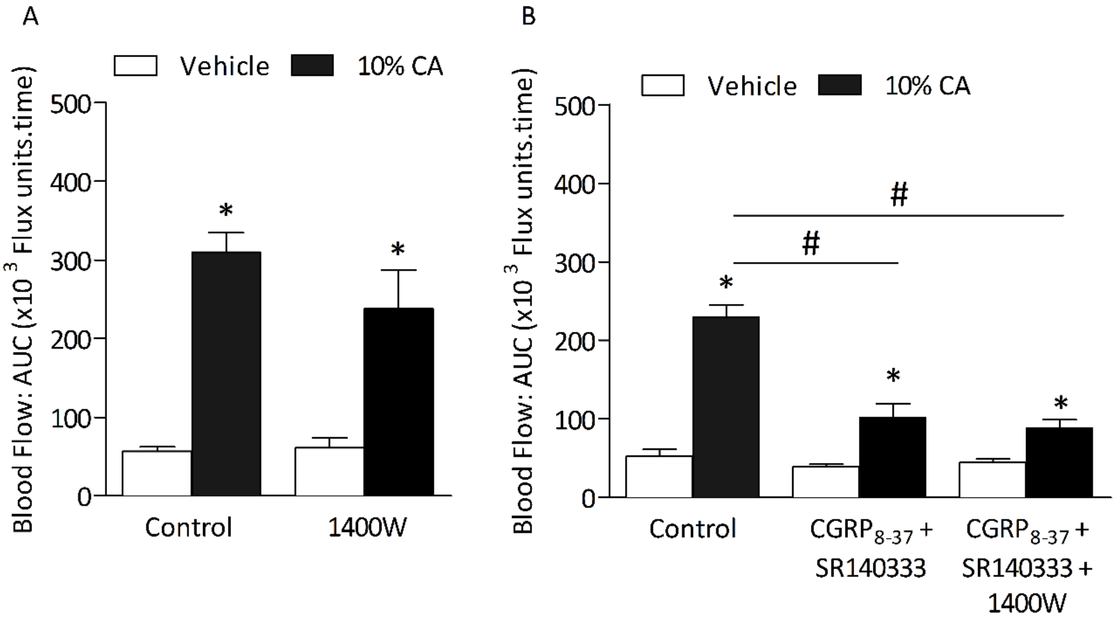

Figure S4 Cinnamaldehyde (CA)-induced vasodilatation is not dependent on iNOS-derived nitric oxide. Blood flow was measured in response to topical application of 20µl of cinnamaldehyde (10% CA) and vehicle (10% DMSO in ethanol) in the anaesthetised mouse ear. Results were recorded over 30 min and analysed as area under the curve (AUC). Group mean data for CA-induced vasodilatation in WT mice pre-treated with (***A***) the selective iNOS inhibitor 1400W alone (3mg kg^{-1}, n=9) or control (saline, n=9) and (B) a combination of 1400W (3mg kg^{-1}) with $CGRP_{8-37}$ (400nmol kg^{-1}) and SR140333 (480nmol kg^{-1}) or control (saline, n=5-7). All errors indicate SEM. *p<0.05 vs vehicle-treated, #p<0.05 vs CA-treated ears of WT mice (2-WAY ANOVA, Bonferroni *post hoc* test).

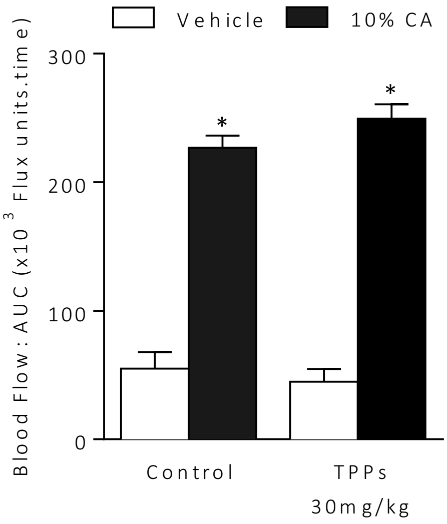

Figure S5 Effects of Tetraphenylporphinesulfonate (TPPS) on cinnamaldehyde (CA)-induced vasodilatation. Blood flow was measured in response to topical application of 20µl of cinnamaldehyde (10% CA) and vehicle (10% DMSO in ethanol) in the anaesthetised mouse ear. Results were recorded over 30 min and analysed as area under the curve (AUC). Group mean data for CA-induced vasodilatation in WT mice pre-treated with TPPS (30mg kg^{-1}, n=5) or control (saline, n=5). All errors indicate SEM. *$p<0.05$ vs vehicle-treated ears of WT mice (2-WAY ANOVA, Bonferroni *post hoc* test).

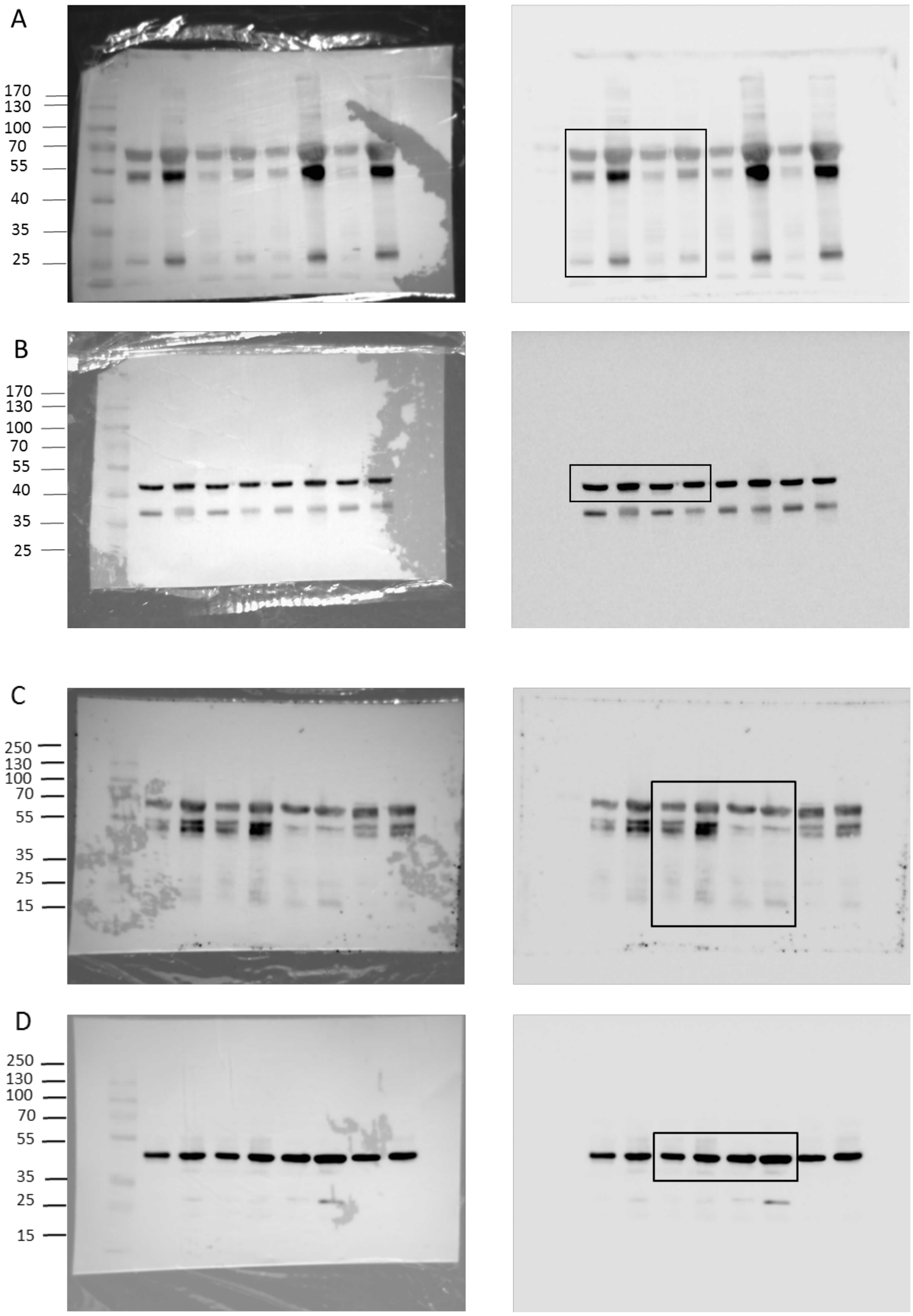

Figure S6: Uncropped immunoblots for Figure 5C-D displayed in the main figures. Immunoblots are developed using Syngene gel doc digital dark room system. A digital image of the membrane is acquired, following which, the immunoblot is developed to reveal the probed protein bands (kDa). ***(A)*** A merged image of the captured membrane and developed nitrotyrosine immunoblot (left panel), and uncropped immunoblot for nitrotyrosine for Figure 5C (right panel). ***(B)*** A merged image of the captured membrane and developed β-actin

immunoblot (left panel), and uncropped immunoblot for β-actin for Figure 5C (right panel) for vehicle and cinnamaldehyde-treated tissue samples in WT mice pre-treated with FeTPPS (30mg kg^{-1}) or control. ***(C)*** A merged image of the captured membrane and developed nitrotyrosine immunoblot (left panel), and uncropped immunoblot for nitrotyrosine for Figure 5D (right panel). ***(D)*** A merged image of the captured membrane and developed β-actin immunoblot (left panel), and uncropped immunoblot for β-actin for Figure 5D (right panel) for vehicle and cinnamaldehyde-treated tissue samples in TRPA1 WT and KO mice. Boxed areas indicate the cropped regions displayed in Figure 5C-D.
